# Correction: *In situ* monitoring of functional activity of extracellular matrix stiffness-dependent multidrug resistance protein 1 using scanning electrochemical microscopy

**DOI:** 10.1039/d2sc90187k

**Published:** 2022-09-16

**Authors:** Shuake Kuermanbayi, Yaowei Yang, Yuxiang Zhao, Yabei Li, Le Wang, Jin Yang, Yan Zhou, Feng Xu, Fei Li

**Affiliations:** The Key Laboratory of Biomedical Information Engineering of Ministry of Education, School of Life Science and Technology, Xi'an Jiaotong University 710049 Xi'an Shaanxi China fengxu@mail.xjtu.edu.cn feili@mail.xjtu.edu.cn; Bioinspired Engineering and Biomechanics Center (BEBC), Xi'an Jiaotong University 710049 Xi'an Shaanxi China; School of Chemistry, Xi'an Jiaotong University 710049 Xi'an Shaanxi China; Department of Oncology, The First Affiliated Hospital of Medical College, Xi'an Jiaotong University 710049 Xi'an Shaanxi China

## Abstract

Correction for ‘*In situ* monitoring of functional activity of extracellular matrix stiffness-dependent multidrug resistance protein 1 using scanning electrochemical microscopy’ by Shuake Kuermanbayi *et al.*, *Chem. Sci.*, 2022, https://doi.org/10.1039/d2sc02708a.

The authors regret that an incorrect version of Fig. 5f was included in the original article. This error does not affect the conclusions of the original article as the correct Fig. 5f also proves that there is no significant difference in the mRNA levels of MRP1 in the MCF-7 cells on the PA gels with three stiffness. The correct version of Fig. 5 is presented below.

**Fig. 1:**
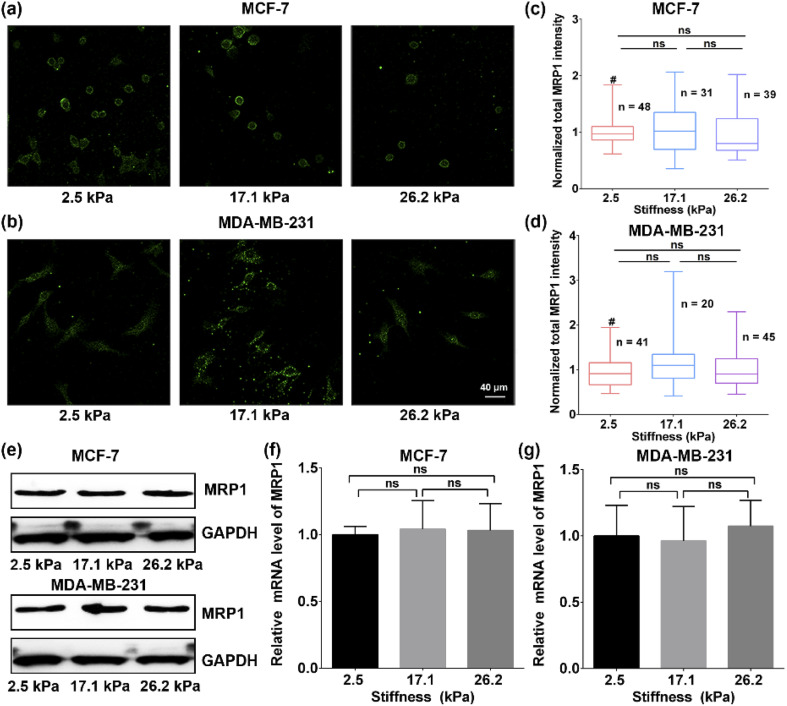
(a and b) Immunofluorescence images and (c and d) the normalized total MRP1 intensities of (a and c) MCF cells and (b and d) MDA-MB-231 cells on the PA gels with stiffness of 2.5, 17.1 and 26.2 kPa, respectively (scale bar: 40 μm). (e) Western blot analysis of the MRP1 expressions of the MCF-7 and MDA-MB-231 cells on the PA gels with stiffness of 2.5, 17.1 and 26.2 kPa, respectively. (f and g) The relative MRP1 mRNA expressions in (f) the MCF-7 cells and (g) the MDA-MB-231 cells on the PA gels with stiffness of 2.5, 17.1 and 26.2 kPa, respectively.

The Royal Society of Chemistry apologises for these errors and any consequent inconvenience to authors and readers.

## Supplementary Material

